# Functional analysis of transcription factor binding sites in human promoters

**DOI:** 10.1186/gb-2012-13-9-r50

**Published:** 2012-09-05

**Authors:** Troy W Whitfield, Jie Wang, Patrick J Collins, E Christopher Partridge, Shelley Force Aldred, Nathan D Trinklein, Richard M Myers, Zhiping Weng

**Affiliations:** 1 Program in Bioinformatics and Integrative Biology and Department of Biochemistry and Molecular Pharmacology, University of Massachusetts Medical School, Worcester, MA 01605, USA; 2 SwitchGear Genomics, Menlo Park, CA 94025, USA; 3 HudsonAlpha Institute for Biotechnology, Hunstville, AL 35806, USA

## Abstract

**Background:**

The binding of transcription factors to specific locations in the genome is integral to the orchestration of transcriptional regulation in cells. To characterize transcription factor binding site function on a large scale, we predicted and mutagenized 455 binding sites in human promoters. We carried out functional tests on these sites in four different immortalized human cell lines using transient transfections with a luciferase reporter assay, primarily for the transcription factors CTCF, GABP, GATA2, E2F, STAT, and YY1.

**Results:**

In each cell line, between 36% and 49% of binding sites made a functional contribution to the promoter activity; the overall rate for observing function in any of the cell lines was 70%. Transcription factor binding resulted in transcriptional repression in more than a third of functional sites. When compared with predicted binding sites whose function was not experimentally verified, the functional binding sites had higher conservation and were located closer to transcriptional start sites (TSSs). Among functional sites, repressive sites tended to be located further from TSSs than were activating sites. Our data provide significant insight into the functional characteristics of YY1 binding sites, most notably the detection of distinct activating and repressing classes of YY1 binding sites. Repressing sites were located closer to, and often overlapped with, translational start sites and presented a distinctive variation on the canonical YY1 binding motif.

**Conclusions:**

The genomic properties that we found to associate with functional TF binding sites on promoters -- conservation, TSS proximity, motifs and their variations -- point the way to improved accuracy in future TFBS predictions.

## Background

The interaction between transcription factor (TF) proteins and DNA is elementary to the regulation of transcription, a coordinated process that responds to environmental factors to achieve temporal and tissue specificity [1,2]. Therefore, the ability to predict and identify TF binding sites throughout genomes is integral to understanding the details of gene regulation and for inferring regulatory networks [3]. The list of environmental factors affecting the transcriptional regulation by a TF includes the binding of additional TFs [4-6], histone modifications, and chromatin remodeling.

Due to the importance of identifying transcription factor binding sites (TFBSs), efforts to identify these sites computationally are ongoing and intense [3,6-12]. The most basic elements used for identifying TF binding sites from sequences are the characteristic binding properties for each TF, comprising the width of DNA binding site and the nucleotide preferences at each position. These properties are quantitatively described by a position weight matrix (PWM) [13] and can be deduced from aligning a set of DNA sequences that are experimentally known to bind the TF. Used on their own, single PWMs, or motifs, typically predict a binding site for every 5 kb of DNA. In the human genome, we know that the vast majority of these predicted sites do not function in the cell. While they can accurately predict *in vitro *binding [14], position weight matrices represent the *in vivo *reality more accurately when used in concert with additional knowledge. For example, phylogenetic footprinting [15] and cooperativity between transcription factors [4-6] have been shown to be a useful supplement to known PWMs.

A major challenge in the effort to map TF binding sites comprehensively is to complement TFBS predictions with a high-throughput experimental approach that directly validates the functional contribution made by transcriptional regulatory motifs [11]. In addition to validating computationally predicted TF binding sites, functional tests reveal whether a given binding event has the effect of activating or repressing transcription. Such measured functional outcomes of TF binding have direct implications for biological networks, cell differentiation, and disease and should inform next-generation algorithms for identification of TF binding sites.

Taking advantage of data generated by the ENCODE Consortium [16-18], we carried out a large-scale systematic functional analysis, at base-pair resolution, of predicted TF binding sites in four immortalized human cell lines by performing transient transfection assays on promoters [19-22]. To predict TF binding sites, we used high-throughput chromatin immunoprecipitation with sequencing (ChIP-seq) data that have been collected on a vast scale by the ENCODE Consortium. Although ChIP-seq data are a powerful way to map regulatory relationships, they do not resolve TF-DNA binding footprints at base-pair resolution. Typical binding regions determined from ChIP-seq data are on the order of hundreds of base pairs in size. The direct application of single motifs, represented as PWMs [13], to scan the sequences is known to be high resolution but suffers from a high false-positive rate [10]. We have combined ChIP-seq data generated by members of the ENCODE Consortium [16-18] with PWM searches using known motifs [23,24] to generate a set of predicted TF binding sites (see Materials and methods section for details). The transient transfection promoter activity assay fuses a putative promoter sequence with a reporter gene (here, luciferase) in a plasmid construct. The recombinant plasmid is transfected into mammalian tissue culture cells, and the activity of the regulatory segment is inferred from the amount of reporter gene activity that occurs. This assay connects the promoter sequence with measured transcriptional activity. Our investigation was focused on six transcription factors: CTCF, GABP, GATA2, E2F proteins, STAT proteins, and YY1.

The CCCTC binding factor (CTCF), a DNA-binding protein with 11 zinc finger domains, is the most thoroughly characterized insulator-binding protein in humans [25-27]. While CTCF has been shown to function as an enhancer blocker [28], it is also known to repress [29] and activate [30] transcriptional activity. In addition, CTCF has been shown to play an unusual role in positioning nucleosomes [31] and to be important for global chromatin organization [27]. Given its diversity of function, CTCF, originally described as a 'multivalent factor', [32] appears to have a special status among transcription factors [26]. The GA-binding protein (GABP) is an ETS family transcription factor that functions as a heterodimer composed of the DNA-binding GABP*α *and transcriptionally activating GABP*β *subunits [33]. GABP is known to play an essential role in cell-cycle progression [34], T cell development [35] and early mouse embryogenesis [36]. As a transcriptional regulator, GABP is known to be strongly activating, with tandem GABP binding sites able to initiate transcription in the absence of other cis elements [37]. GATA proteins form a family of six regulatory proteins, each with a highly conserved DNA-binding domain containing two zinc fingers that target the DNA sequence (A/T)GATA(A/G) [38,39]. The GATA proteins are divided into two subfamilies based on their expression patterns [40,41]. The subfamily composed of GATA1, -2, and -3 had been categorized as the 'hematopoietic' group [41] due to their regulation of differentiation-specific genes in hematopoietic stem cells. GATA1 is expressed in cells from the myeloid lineage, including erythroid cells, eosinophils, mast cells, megakaryocytes, and dendritic cells [42,43], while GATA2 is expressed in a wider variety of tissues, including hematopoietic progenitors, erythroid cells, mast cells, megakaryocytes, adipocytes [44], endothelial cells, and embryonic brain cells [42,45,46]. GATA3 is highly expressed in embryonic brain cells and T lymphoid cells but has been found in other tissues [45,47]. GATA4, -5, and -6 have been categorized as the 'endodermal' group [41] because they are expressed (in overlapping patterns) in several endoderm-derived tissues including the heart, gut, lung, and liver [48]; they may be involved in regulating cardiogenesis and the differentiation of gut epithelium [42].

The first member of the E2 factor (E2F) transcription factor family was identified as a protein that activates the adenoviral E2 gene by binding its promoter [49]. As a group, the E2F proteins are important regulators of cell cycle and DNA synthesis [50-54]. Eight members of this family have been identified based upon sequence homology, E2F1-E2F8 [53,54]. The regulatory functions of E2F proteins are mediated by the Rb family of 'pocket proteins': retinoblastoma protein (pRb), p107, and p130 [51-53,55,56]. E2F6-8 lack the Rb protein binding domain [57], while E2F4 binds to all members of the Rb family; E2F1-3 bind only to pRB; E2F5 binds to p130. The functional classification of E2F family members aligns with their respective binding specifiicities for pocket proteins: E2F1-3 are considered transcriptional activators (their overexpression can drive quiescent cells into S-phase [52]); E2F4 and E2F5 are regarded mainly as repressors [51,57], although recent analysis of E2F4 overexpression in HeLa cells reveals many upregulated E2F4 target genes [58]. DNA binding of the E2F6-8 proteins has been associated with transcriptional repression [57]. All members of the E2F family share a conserved DNA-binding domain [59,60] and have been reported to bind the same TTT(C/G)(C/G)CGC motif *in vitro *[54].

The signal transducer and activator of transcription (STAT) proteins comprise a family of latent cytoplasmic signal-dependent transcription factors [61]. Cytoplasmic STATs can be activated by a wide variety of extracellular signals such as cytokines, growth factors. and hormones that bind to specific cell surface receptors, leading to STAT phosphorylation on a single tyrosine located near residue 700 [61,62]. STAT-phosphorylating receptors include Janus kinases and receptor tyrosine kinases (TKs). Even without ligand-binding events, however, STAT proteins can be phosphorylated by non-receptor TKs [63,64]. Upon phosphorylation, STAT proteins form homo- or heterodimers via interactions between their respective Src homology 2 phophotyrosine-binding domains [61,64,65]. STAT dimers then translocate to the nucleus and bind to their target DNA loci. Seven mammalian STAT proteins, exhibiting differential response to extracellular signals, have been identified to date: STAT1-4, STAT5A, STAT5B, and STAT6. Of these, STAT1, STAT3-4, STAT5A, STAT5B, and STAT6 form homodimers; STAT1:STAT2, STAT1:STAT3, and STAT5A:STAT5B heterodimers also form, depending upon the nature and concentration of signaling moieties [61,62,64,65]. STAT proteins regulate the expression of genes that are important for immune defense, in ammation, antiviral response, differentiation, proliferation, and apoptosis [61,66]. STAT homodimers bind to so-called IFN-*γ *stimulated gene response (GAS) DNA elements (a palindrome, TTN5-6AA) [61,64,65]. STAT2 is the only STAT protein that does not bind GAS elements as a homodimer; STAT1:STAT2 heterodimers associate with p48 (also known as IRF9) to form the ISGF3 transcription factor complex, which recognizes IFN-stimulated response element (ISRE) DNA sequences (AGTTTNNNTTTCC) [65,67-71]. Our mutagenesis experiments were focused on binding sites for (STAT1:STAT1) homodimers recognizing GAS sequences.

Yin Yang 1 (YY1) is a ubiquitously expressed transcription factor whose name derives from its ability to function as an activator, repressor, or initiator of transcription, depending upon additional regulatory factors [72]: when first identified, YY1 was found to repress transcription of the adeno-associated virus when bound to the P5 promoter region but to activate its transcription in the presence of the adenovirus E1A protein [73]. YY1 is found in both invertebrates and vertebrates and is highly conserved. Placental mammals have two YY1 paralogues, YY2 and reduced expression 1 (REX1), which have been shown to result from retrotransposition events early in the mammalian lineage [74]. Whereas YY2 binds to YY1 motifs (AANATGGN(C/G) [75,76]) with greatly reduced affinity [74], REX1 recognizes motifs that are divergent from those of YY1 [74]. Based upon these findings, we expect that our predicted YY1 binding sites will predominantly be recognized by YY1, rather than its paralogues. It has been reported from motif analysis of high-throughput DNA binding data (ChIP-chip) that YY1 binding sites may be categorized into two distinct classes: one class with binding sites located downstream of the transcriptional start site (TSS), overlapping with translational start sites and another class upstream, or frequently atop, the TSS [77]; in this work, we find that these two classes map onto functional categories, with the former being associated with transcriptional repression and the latter with activation.

To better understand the functional consequences of TF binding, both globally and as it relates to the specific transcription factors listed above, we analyzed the results of transient transfection promoter activity assays carried out in K562, HCT116, HT1080, and HepG2 cell lines. In each assay, we compared the activity of the wild-type promoter construct with that of a mutant promoter construct in which the predicted TF binding site was abolished (see Materials and methods section). We observed a functional contribution of predicted TF binding sites to promoter activity at a rate of 49% in K562 cells, 38% in HCT116 cells, 36% in HT1080 cells, and 39% in HepG2 cells. Our data show that, compared with TF binding sites where function was not observed, sites that were functionally verified were more conserved and located closer to the TSS. We discovered that more than one-third of the experimentally verified TF binding sites repressed transcriptional activity when bound by a TF, and we carried out similar analyses to discover the patterns that govern the relationship between TF binding and activation versus repression of transcription.

## Results and discussion

As described in the Materials and methods section, high-throughput ChIP-seq data were used in conjunction with known specificities (PWMs) to identify putative TF binding sites on human promoters. The resulting set of promoters was then mutagenized, and transient transfection promoter activity assays were carried out on both wild-type and mutant constructs in order to detect significant differences in transcriptional activity. The mutations were chosen to abolish TF binding by mutating as many as five nucleotides in the most informative (that is, making the greatest contribution to the TF-DNA binding free energy) positions.

For our purposes, the transient transfection approach has the benefit that it measures the function of a specified DNA fragment, thereby making a direct connection between sequence and function. Another aspect of the method, however, is that it removes the promoter from its native environment. This displacement implies that long-range regulatory elements are largely missing. Plasmids are chromatinized when transfected, yet their chromatin structure differs from that of the endogenous genes and promoters. In spite of this departure in chromatin structure, transient transfection reporter assays often yield tissue-specific information [21,22].

We performed transfection experiments for each promoter (wild-type or mutant) in three biological replicates and three technical replicates per biological replicate. We analyzed the resulting reporter data using a *t *test to detect mutant transcriptional activity that was significantly different from that of the wild type. Binding sites in which the mutated version had FDR < 0.025 (after correcting for multiple testing using the Benjamini-Hochberg rule) were taken to be functionally verified. The verified mutated binding sites that had lower average luminosities than their corresponding wild type indicate that these sites serve to activate transcription, whereas mutated sites with higher luminosities than the wild type are indicative of a repressing effect on transcription.

**Figure 1:**
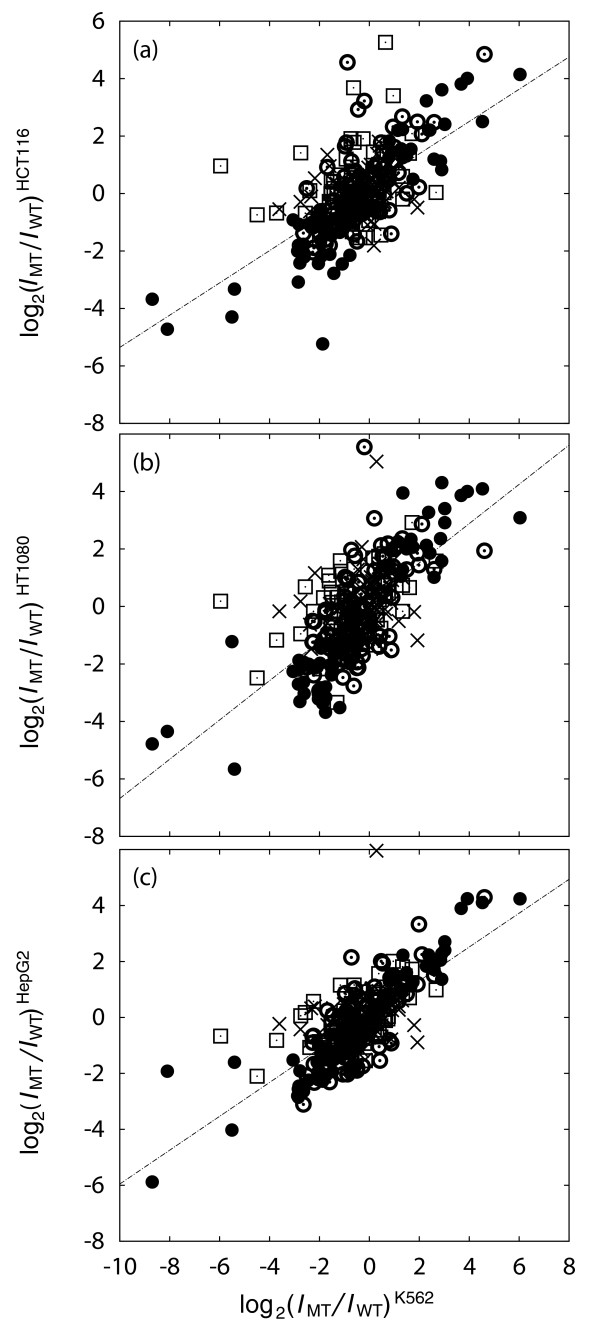
**Pairwise scatter plots for luciferase signals**. Plotted is |log_2_(*I*_MT_/*I*_WT_)|, where *I*_MT _and *I*_WT _are the mutant and wild-type normalized luminosities, respectively, in four cell lines (K562, HCT116, HT1080 and HepG2). Experiments plotted are those where TFBS function was validated in all four cell lines (bullet), three cell lines (open circle with middle dot), two cell lines (open square with middle dot) and one cell line (cross). The Spearman correlation coefficients for experiments carried out in K562 cells with those in HCT116 cells **(a)**, HT1080 cells **(b)**, and HepG2 cells **(c) **are 0.57, 0.64 and 0.65, respectively.

The results were consistent across the different cell lines, as shown in Figure 1, where the logarithm of the ratio of mutant to wild type luciferase signal is plotted for pairs of cell lines. The intensities of luciferase luminosities were normalized on each plate using all signals, including four positive and four negative control transfections. Note that the linear relationship shown in Figure 1 between the measured transcriptional effect of TFBS disruption in one cell line with that in another cell line implies an underlying dependence on TF concentration: an *n*-fold effect in one cell line is consistently matched to an *m*-fold effect in another cell line. When we carried out linear fitting on for individual transcription factors, we determined that the slopes (that is, *n*/*m*) were different (within the error from least-squares fitting) for different TFs (Figure S1 in Additional file 1). In order to make a more direct connection between measured luciferase signals and *in vivo *TF concentration, we compared measured wild-type luminosities in different cell lines (Figure S2 in Additional file 1) and ENCODE Consortium [16-18] RNA sequencing data (Figure S3 in Additional file 1), finding a (Pearson) correlation coefficient of 0.59.

The Venn diagram in Figure S4 in Additional file 1 summarizes the results of our functional tests in four cell lines. In total, we assayed 455 putative TF binding sites across all cell lines and found that 135 sites were not functionally verified in any cell line. The numbers in parentheses in Figure S4 in Additional file 1, (*n*-activating, *n*-repressing), are for binding sites that were consistently either activating or repressing across all cell types in which they were functionally verified. For example, in Figure S4 in Additional file 1, there are 63 predicted TF binding sites that were functional in all four cell lines, 39 of which were associated with ubiquitous activation of transcription and 23 with ubiquitous repression (here, and throughout the remainder of the paper, we de ne 'ubiquitous' to mean across all four cell lines in our study). The remaining ubiquitously verified TFBS (for YY1, bound at the translational start site of the Metaxin-3 gene) presented cell line-dependent transcriptional activity: activating effects were observed in the K562, HT1080, and HepG2 cell lines, and repression was observed in the HCT116 cell line. The determination of a transcriptionally activating versus repressing function of TF binding is not possible with experimental methods such as ChIP-seq nor by most computational approaches: these functional data provide new and important information for understanding gene regulation at levels of both individual genes and networks. Table 1 summarizes our data according to the TFs in our assays. The majority of the sites in our tests are bound by six TFs: CTCF, E2F family proteins, GABP, GATA2, STAT1, and YY1 (that is, the TFBS sequences appear to be directly occupied by these factors; see Materials and methods section). These TFs have varying rates of being functional in at least one cell line, with CTCF, E2F family proteins, GABP, and GATA2 all exhibiting a functional verification rate of approximately 60%, while STAT1 and YY1 had their function verified at rates of 78% and 88%, respectively. However, compared with the other factors in our experiments, CTCF and GATA2 had a much lower fraction of functional sites across all four cell lines. In the case of GATA2, this observed lower rate of ubiquitous function may be due to the varying expression levels of GATA family proteins in different cell lines. For example, it has been reported that HepG2 cells do not express GATA2 or GATA3 [78] but do express GATA4 [79] (these observations are broadly confirmed by the ENCODE Consortium [16-18] RNA sequencing data reported in Table S2 in Additional file 1). GATA6 is highly expressed in colon cancer epithelial cells [48], such as HCT-116. Since CTCF is broadly expressed, the relatively low rate of ubiquitous function across all four cell lines may be due to combinatorial interactions with other TFs. For example, in Table 2, we note that promoters with a functionally verified CTCF binding site were significantly enriched in AP-2, E2F4, GABP, NF-Y, and Pax5 motifs.

**Table 1:** Summary of functional tests of 466 predicted TF binding sites in four human cell lines

TF	**Func**.	Tested	**Ubq.Func**.	**Ubq. Act**.	**Ubq. Rep**.	Func. K562	Func. HCT116	Func HT1080	Func HepG2	PWM	AUC
CTCF	104	168	9	9	0	62	52	49	53	Ref. [31]	0.84
E2F4	7	12	0	0	0	3	3	3	0	E2F:4 M00739	0.83
E2F6	2	3	0	0	0	1	0	1	0	E2F:1 M00938	0.78
EGR1	1	2	0	0	0	0	1	0	0	Egr:3 M00245	0.76
GABP	7	11	4	4	0	5	5	6	5	Ref. [103]	0.77
GATA1	4	4	1	1	0	4	4	1	1	GATA:1 M00128	0.69
GATA2	47	80	4	3	1	36	20	18	14	GATA:2 M00348	0.81
JUND	3	3	1	1	0	2	2	1	3	CREBP1 M00041	0.65
MAX	3	3	1	0	1	2	2	2	2	cMycMax M00118	0.77
STAT1	54	69	16	11	5	41	27	29	39	STAT1 M00224	0.74
USF1	2	2	1	1	0	2	2	2	1	USF M00121	0.86
YY1	86	98	26	9	16	63	56	53	58	Ref. [103]	0.82

Total	320	455	63	39	23	221	174	165	176		

For each TF that was part of our functional study, columns list the number of functional verifications in at least one cell line, number of TFBSs tested, number of ubiquitously functional TFBSs, number of ubiquitously activating TFBSs, number of ubiquitously repressing TFBSs, number of TFBSs functionally verified in each cell line, the source of PWM used and the corresponding AUC when applied ChIP-seq data sets. The PWMs are shown as motif logos in Table S1 of Additional file 1.

**Table 2:** Analysis of over- and underrepresented secondary motifs on promoters

TF	TF2	*p ***value**
CTCF	AP-2	< 0.001
	E2F4	< 0.001
	GABP	0.031
	LBP-1	0.999
	NF-Y	< 0.001
	Pax5	0.046
STAT1	AP-1	< 0.001

Secondary motifs (TF2 column) over- and underrepresented on promoters with functional TFBSs (TF column) versus promoters with nonfunctional TFBSs (TF column).

None of the binding sites tested for E2F4 and E2F6 showed ubiquitous function across all four cell types. Of the 12 E2F4 binding sites that were tested, 7 showed function in at least one cell line: three binding events lead to activation of transcription, and four lead to transcriptional repression. Of the three E2F6 binding sites that were tested, two displayed function in at least one cell type, leading to transcriptional repression in both cases. Although the total number of E2F family binding sites tested was relatively few, these results are in line with the current understanding of the regulatory modes for E2F4 and E2F6, with the former leading to both activation and repression of gene expression [58] and the latter being exclusively repressing [57].

Among the ubiquitously functional sites, a majority of those for CTCF, GABP, GATA2, and STAT1 have an activating effect, but only one-third of YY1 sites are activating. This result is perhaps a surprising one for CTCF, which is generally regarded as a chromatin organizer [27] and insulator-binding protein [25-27], but is also known to act both as a repressor [32] and as an activator [30]. If CTCF is acting as an insulator, the implication would be that disruption of the CTCF binding site leads to a decreased transcriptional activity via repressive elements on the same promoter that are no longer under its influence. The comparison between CTCF and YY1 is shown in Figure S5 in Additional file 1, where it is clear that some YY1 sites have strong repressing effects and where our ubiquitously functional CTCF sites have strong activating effects. We show below that the repressive YY1 sites are distinguished by their location relative to the translational start site. Table 3 lists the genes whose expression was ubiquitously activated and repressed, respectively in our four cell lines, according to TF.

**Table 3:** Summary of genes regulated by ubiquitously functional TFBSs for five TFs: CTCF, GABP, GATA2, STAT1, and YY1

TF	Ubiquitously activated	Ubiquitously repressed
CTCF	AL645504.2	
	ANKRD46	
	BICD2	
	C17orf81	
	CEP135	
	CRYAA	
	EGLN2	
	POMT2	
	TSFM	
GABP	GART*^a^*	
	PSMB4*^a^*	
	SYNJ1*^a^*	
	ZNF259*^a^*	
GATA2	CTSH	CCM2
	PLSCR2	
	TNFAIP8L1	
STAT1	ATG4C	HCFC1
	DCLRE1C	RPS24
	DIMT1L	TMED5
	ELP3	XXbac-
		BPG116M5.1
	GSTK1	ZNF367
	IRF7*^b^*	
	IRF9*^b^*	
	KIF2A	
	MTMR9	
	NMI	
	SBNO2	
YY1	COQ5*^cd^*	AC091153.1
	CPNE1	ATP5O
	CPSF2*^cd^*	BIRC6*^d^*
	CR613718	CAPZA2
	IP6K2*^a^*	CXorf26
	NARS*^ac^*	DKFZp434H247
	PAK4^d^	EFHA1
	PSMB4*^ac^*	MRPS10*^c^*
	UBR5	MRPS18B*^acd^*
		NUP160
		OXCT1
		PSMD8*^ac^*
		SNX27
		SNX3*^ad^*
		SRP68*^ad^*
		TNKS

A composite of RefSeq [104], UCSC known genes [105] and GENCODE [106] annotations were used. The GREAT gene annotation tool [107] was used to compare our data with results from published experiments: *a *targets of CREB, identified by ChIP-chip in HEK293T cells in three different time points after forskolin stimulation [81], *b *genes upregulated by tamoxifen [108] in HMEC-E6 cells, genes upregulated in Jurkat cells by IFN-*α *and IFN-*β *but not by overexpression of a constitutively active form of IRF3 [109], c targets of YY1 identified by Chip-chip [77], *d *targets of HNF4*α *identified by Chip-chip in hepatocytes from TF targets.

Of the seven GABP binding sites in which we observed a functional effect on transcription, all binding events had an activating effect; the four GABP binding sites with ubiquitous function across each of our cell lines were activating (see Table 3). This observation is consistent with previous evidence for GABP as a general activator [37,80]. The genes whose transcription is ubiquitously activated by GABP binding are listed in Table 3. All of these genes are known targets of the cAMP-response element binding protein (CREB) [81], a known co-activator of GABP [82,83].

Ubiquitously activated targets of STAT1 binding listed in Table 3 include IRF7 and IRF9, both of which are members of the interferon regulatory factor family, proteins involved in immune response. IRF7 and IRF9 are both known to respond to extracellular signaling (see Table 3). IRF7 is critical to the type-I interferon (INF-*α*/*β*) response to viral infection [84], while IRF9 (also known as p48) forms the ISGF3 transcription factor complex with the STAT1:STAT2 heterodimer to bind ISRE DNA sequences. Ubiquitously functional targets of YY1 binding are listed in Table 3 and include genes known not only from previous ChIP experiments to detect YY1 binding [77], but also those to detect HNF4*α *[85] and CREB [81] binding. This binding is consistent with previous analysis of ChIP-chip data for YY1, which has revealed a small but statistically significant enrichment of CREB binding sites within experimentally determined YY1 binding regions [77]. Moreover, HNF4*α *is a known transcriptional co-activator for the CREB-binding protein. Based on these earlier findings, it is reasonable to expect overlapping targets for YY1, HNF4*α *, and CREB among our set of functional YY1 binding sites.

We compared the fold change in reporter signal, |log_2 _(*I*_MT_γ*I*_WT_)|, between different groups of TF binding sites defined in Figure S6 in Additional file 1, where *I*_MT _and *I*_WT _are the mutant and wild-type normalized luminosities, respectively. As can be seen in Figure S6 in Additional file 1, TF binding sites that were functionally verified across all four cell lines showed the highest magnitude in fold change, statistically different from sites that were not ubiquitously functional (*p *< 2 10^-16^). While the observed pattern of increasing fold change being associated with functional verification in a greater number of cell lines may be biologically important, it may also be that we were able to detect ubiquitous function more readily in the binding sites that led to the strongest effects on transcription.

It is known that human promoters cluster into two groups based upon normalized CpG content: the high CpG promoters that are associated with strong expression across a broad range of cell types and the low CpG promoters that are associated with weaker but tissue-specific expression [22]. To determine the effect of CpG content in the wild-type promoter on whether a site is functional, we compared the CpG content between the promoters with one or more TFBSs verified in all four cell lines with those having no functionally verified TFBSs (*p *= 0.29). We also compared the promoters with ubiquitously functional TFBSs to those having a TFBS that was functionally verified in only one cell line or else not functional (*p *= 0.23). In neither comparison did we observe a significant difference in normalized CpG content.

Groups of binding sites for the same TF, so-called homotypic clusters of TFBSs (HCTs), have been computationally detected in the human genome on the basis of known PWMs [86]. It has been suggested that such homotypic clusters may offer mechanistic advantages, or simple functional redundancy in transcriptional regulation. Enrichment in HCTs has been found in promoters and enhancers [86]. In the context of our tests of TFBS function, one might expect the presence of HCTs to impact the transcriptional response to the disruption of a single TFBS, with additional binding sites for the same TF compensating for its loss. Accordingly, we searched our promoter set for HCTs of the assayed TFs. For each of our putative TF binding sites, we re-scanned its promoter using the same motif (see Table S1 in Additional file 1 and Table 1) and score threshold as was used in our predictions. From this re-scanning, we detected up to three instances of homotypic TFBSs on a single promoter. A large majority of our promoters, however, contained only a single homotypic TFBS. For example, of the 168 CTCF binding sites that were tested (see Table 1), 135 were on promoters with a unique instance, 32 were on promoters with two instances, and 1 was on a promoter containing three instances. For YY1, we functionally tested binding sites: 88 promoters with a single instance of YY1 binding, 6 with a pair of instances, and 4 with three instances. We compared the number of homotypic TFBS instances per promoter between functional classes of TFBSs, observing a general trend of higher verification rates for promoters with fewer homotypic TFBSs. This observation, however, was not statistically significant: *p *< 0.78 when comparing promoters having ubiquitously functional CTCF binding sites to those with ubiquitously unverified predicted CTCF binding sites. When this same comparison is made for promoters with predicted YY1 binding sites, *p *< 0.99. Functional classes of TFBSs could not be distinguished on the basis of the number of homotypic binding sites on the same promoter for any of the TFs in our study. The response in transcriptional activity implied by multiple homotypic TFBSs on a given promoter likely depends upon the details of homotypic TFBS distribution, such as the conservation at each site, the distance between instances, and the presence of intervening heterotypic TFBSs.

### Functional analysis of transcription factor co-localization on promoters

In Table 2, we list secondary TF motifs whose overrepresentation (or underrepresentation) on promoters containing binding sites for CTCF and STAT1, respectively, can be related to a functional outcome. The motifs listed in the 'TF2' column of Table 2 are statistically overrepresented (or underrepresented) on promoters with a functional binding site for transcription factors listed in the 'TF' column (that is, CTCF and STAT1), relative to promoters with a predicted (CTCF or STAT1) binding site whose function was not verified. As a starting point for our analysis, the secondary motifs (TF2) were constrained to be among those exhibiting statistically significant co-localization based upon an analysis of 490 ENCODE Consortium [16-18] ChIP-seq data sets. From this analysis of ChIP-seq data, reported elsewhere [87], 96 heterotypic motifs were found to be significantly co-localized (the list of TF motifs that co-localize with those in our functional study is presented in Table S3 Additional file 1). Focusing our analysis on motifs that have exhibited co-localization in large-scale data sets has the advantage of adding confidence to our findings and allows us to use high-quality motifs, derived from the ChIP-seq experiments. For each TF with more than 20 predicted binding sites in the present study (to ensure statistically reliable results), we tested for statistical overrepresentation of motifs [9] on promoters with functionally verified (in at least one cell line) TFBSs versus promoters with TFBSs that were not functionally verified. For GATA2 and YY1, we did not observe any overrepresented motifs, in the former case due to the short list of co-localized candidates (see Table S3 in Additional file 1) and in the latter case due to the high rate of TFBS function.

We found that several transcription factors, including AP-2, E2F4, GABP, NF-Y, and Pax5, were overrepresented on promoters with functional CTCF binding sites, compared with promoters whose predicted CTCF binding sites were not functionally verified. Motifs for LBP-1, a transcription factor that regulates genes related to growth and differentiation, are underrepresented on promoters with functional CTCF binding sites, as indicated by its high *p *value (Table 2). Several of the transcription factors that are overrepresented on promoters with functional CTCF binding sites, including AP-2, E2F proteins, and GABP, have recently been reported to be enriched in genomic loci that are constitutively bound by CTCF across multiple tissue types in different species (chicken, mouse, and human) [88]. Our results suggest a transcriptional outcome for the co-localization of CTCF and these motifs on promoters.

We find that the transcription factor AP-1 was overrepresented on promoters with functional STAT1 binding sites, relative to promoters whose predicted STAT1 binding sites were not functionally verified. AP-1 has been identified as a 'potential collaborating' factor for STAT1 in a recent study of microRNA regulation [89].

### YY1 exhibits a variant motif for sites where binding represses transcription

**Figure 2:**
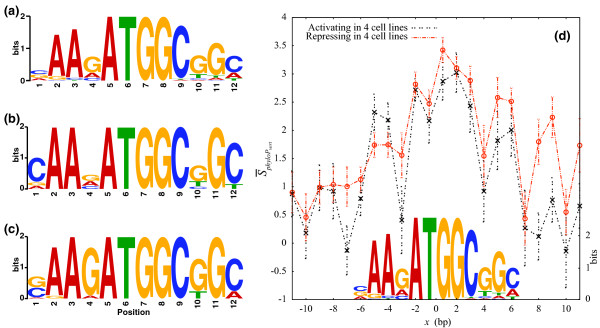
**Characterization of functional YY1 binding sites**. Sequence logo [102] for YY1 binding sites from **(a) **PWM and sites that are functionally **(b) **ubiquitously activating (9 BS) or **(c) **ubiquitously repressive (16 BS) in four human cell lines. In **(d)**, we plot the mean vertebrate phyloP conservation score [90] around functional YY1 binding sites. The mean score, ${\bar{S}}_{phyloP_{\text{vert}}}$, was computed at each base for sites where the binding event ubiquitously activated (black line) or repressed (red line) transcription in all four cell lines. The position weight matrix that was used to predict YY1 binding sites is shown (scale on the right axis).

Among the TF binding sites that were ubiquitously functional, we compared the genomic footprints of sites where binding activated or repressed transcription in all four cell lines. Among the transcription factors we examined (see Table 1), YY1 had the most examples of each case (9 ubiquitously activating and 16 ubiquitously repressing sites). Figure 2 shows the motifs derived from this analysis for YY1. The most striking difference between the YY1 motif for sites where binding is associated with activation (Figure 2b) and those where binding is associated with repression (Figure 2c) occurs at position 4, where the G has greater information content for repressing cases (*p *< 0.012 using a permutation test, see Figure S7 in Additional file 1). The repressive YY1 binding sites are closer to translational start sites than are the activating YY1 binding sites (*p *= 7.7 × 10^-4^). Indeed, 12 of the repressing YY1 binding sites are located directly over the translational start site, whereas only a single activating YY1 binding site is. The mutagenesis experiments reported here elucidate the functional distinction between the different classes of YY1 binding sites that were noted in a previous analysis of DNA binding (ChIP-chip) [77]: the class of YY1 binding sites localized around the translational start site are strongly associated with transcriptional repression, while those localized closer to the TSS are associated with activation.

In Figure 2d, we report the vertebrate phyloP score [90] for each nucleotide, averaged over sites where YY1 binding results in activation or repression of transcription, respectively. Error bars indicate the standard error of the mean. Conservation is generally high for YY1, relative to that for the other transcription factors in our study. At position 4 of the YY1 motif, we observe that mean conservation is lower among the activating sites compared with the repressing sites (*p *< 0.06 using a Wilcoxon rank sum test). We also note that, while both activation- and repression-associated classes of YY1 binding sites show greater conservation over the binding site, relative to flanking regions, the conservation of the repression-associated class is greater than that of the activation-associated class, even beyond the 5' and 3' ends of the YY1 motif.

### Conservation correlates with functional verification rate

**Figure 3:**
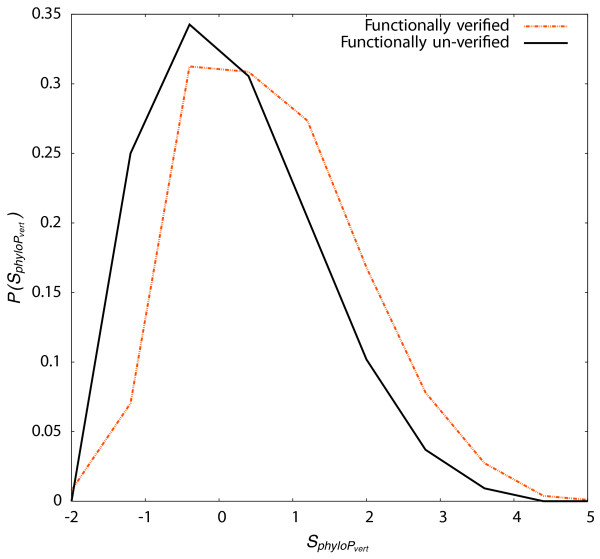
**Conservation differs for functional classes of TF binding sites**. Distributions of normalized vertebrate phyloP score, $S_{phyloP_{\text{vert}}}$ . for TFBSs that were functionally verified in at least one cell line (dashed line) and for TFBSs that were not functionally verified in any cell line (solid line).

Evolutionary constraint is an important factor in discovering functional genomic elements and has been used not only to identify TF binding sites [15,91,92], but also to distinguish real motifs from false positives [93]. For each predicted TFBS, we computed the mean phyloP score [90] for conservation among vertebrates. In Figure 3, we show that TF binding sites that are functionally verified in at least a single cell line are more conserved than those that were not verified in any cell line (*p *= 6.6 × 10^-4^).

### Distance to the TSS correlates with functional verification rate

**Figure 4:**
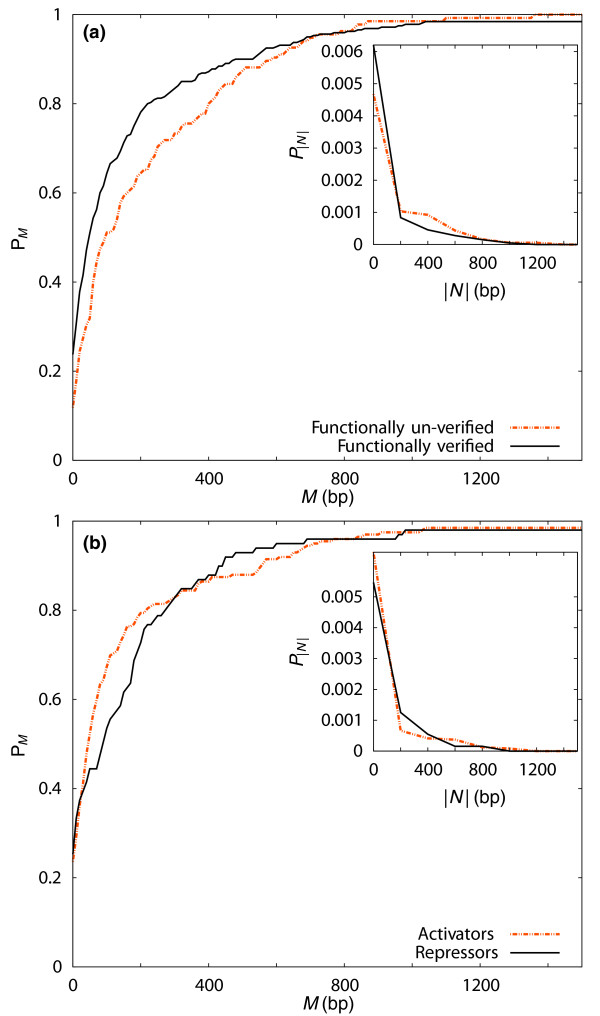
**Using the distance to the TSS to distinguish between TF binding site classes**. Binding sites that were functionally verified or not **(a) **and between activating and repressing TFBSs **(b)**. Here, *P*_|*N*| _= *P*_-*N *_+ *P_N _*is the probability of finding a validated TFBS within |*N*| base pairs of the transcription start site (inset). The cumulative probability, ${\mathbb{P}}_{M}=\sum_{N=0}^{M}P_{\left|N\right|}$, is plotted in the main panels.

In Figure 4a, the distribution of genomic distance between TF binding sites and the TSS is compared between predicted binding sites that were functionally verified in at least one cell line and those that whose function could not be verified. We found that functional TF binding sites tended to be closer to the TSS than TFBSs with unverified function (*p *= 1.8 × 10^-3^).

This result, taken together with our observation of greater conservation among TF binding sites that are functional across many cell lines, is consistent with earlier findings in human promoters [21,94], where it has been noted that much of the constraint appears within 50 bp of the TSS. In Figure 4b, we compared sites where TF binding consistently implied activation of transcription with those where the effect was consistently repressing. We found that activating TF binding sites are significantly closer than repressing TF binding sites to the TSS (*p *= 4.7 × 10^-2^). This observation is not due to the effect of repressing YY1 binding sites being localized on or around the translational start site. Indeed, removing the YY1 binding sites from the overall distributions presented in Figure 4b only increases the significance of the distinction between activating and repressing TFBSs (*p *= 7.5 × 10^-4^). These findings are consistent with those of Cooper *et al*. [21], who detected positive elements on human promoters between 40 and 350 bp away from the TSS, as well as the presence of negative elements from 350 to 1,000 bp upstream of the TSS.

## Conclusions

We have computationally identified 455 putative TF binding sites and functionally tested them in four human cell lines using a transient transfection reporter assay. Overall, 70% of the predicted TF binding sites were functionally verified in at least one of the four cell lines that were used in this study. Of 455 sites, 63 (14%) were verified in all cell lines, 75 (16%) were verified in three cell lines only, 77 (17%) were verified in two cell lines only, 105 were verified in only a single cell line (23%), and 135 (30%) were not functional in any cell line. For each functionally verified TFBS, we were able to determine whether binding led to activated or repressed transcriptional activity in each cell line.

Our large-scale functional tests provide insights into the biology several transcription factors. For CTCF, we determined that functional binding sites were located on promoters for which motifs of the transcription factors AP-2, E2F4, GABP, NF-Y, and Pax5 were overrepresented and LBP-1 was underrepresented. Similarly, the AP-1 motif was overrepresented on promoters with functional STAT1 binding sites. Perhaps more than with any other transcription factor, our experiments shed light on YY1 binding with DNA. Two previously identified classes of YY1 binding sites, characterized by localization at or near the translational start site on the one hand and the TSS on the other, have been related to functional effects on transcription, with the former class associated with transcriptional repression and the latter with activation. Moreover, we have detected a signature variation in binding motifs for functional classes of YY1 binding sites, with the repressing cases showing a preference for G at position 4 of the motif (see Figure 2). It is known that the genomic context of DNA sequences studied using transient transfection represents a departure from the native environment. This departure implies that looping interactions are largely absent, epigenomic features such as histone modifications and even some longer-range cis-regulatory elements may differ from those in the native chromatin. Nevertheless, transient transfection has the important advantage of making a direct link between DNA sequence and function. From analyzing these functional tests, we determined that functional TF binding sites tended to be more conserved and located closer to the TSS than predicted binding sites whose functional impact on promoter activity was not detected. TF binding sites that were ubiquitously functional in all four assayed cell lines were more conserved and located closer to the TSS than sites that were not functionally verified and sites whose function was cell line specific. Moreover, among sites that were ubiquitously functional, those where TF binding led to repression of transcription were located farther from the TSS than those where binding led to activation. Using YY1 as an example, we demonstrated that activating sites and repressive sites can show an evolutionarily conserved difference in a motif position. Taken together, these features can be used to improve the accuracy of TFBS predictions, thereby improving our ability to construct biological networks.

Finally, the approach that we have taken here to identifying and functionally testing TF binding sites can be applied in investigating the functional consequences of variations in sequence and in binding of regulatory elements among individuals [95] and alleles [96]. At present, such variations are characterized at the level of ChIP peaks (hundreds of base pairs) and although such variations are almost certainly associated with determining phenotype, the details remain to be described.

## Materials and methods

### TFBS prediction

We predicted specific binding sites using ChIP-seq data collected primarily in K562 cells (see Table S4 in Additional file 1 for data sets used). For each transcription factor data set, binding regions (or peaks) were called using MACS [97]. For each peak region, a length-matched sequence was randomly selected from the unbound (in the ChIP-seq experiment) regions of the genome; the set of such unbound sequences comprised our background for the purposes of testing and comparing PWMs. After assuming a single (highest scoring) TFBS within each peak of the ChIP-seq signal (and background sequence), we used the POSSUM motif scanner [98] with a library of known PWMs (taken from the TRANSFAC and JASPAR repositories [23,24] and elsewhere [99]) to scan over each data set and compared the scores of the peaks with those from the background sequences. We measured the resulting ability of a PWM to discriminate ChIP-seq peaks from background sequences using the area under (AUC) the receiver operating characteristic curve. An AUC of 0.5 represents the same ability to discriminate as a random classifier, while an AUC of 1 represents perfect discrimination. For each ChIP-seq data set, PWMs were drawn from the TRANSFAC and JASPAR repositories [23,24], such that alternative motifs for the corresponding TF and members of the same TF family were scanned. For each TF upon which we carried out TFBS mutagenesis experiments, the most predictive motif (PWM) is shown in Table 1, along with its corresponding AUC. In a subsequent and separate *de novo *motif discovery analysis [87] of these same ChIP-seq data sets (and others), we confirmed that for each of the TFs appearing in Table 1, the most significant motif could be assigned directly to that TF based on a similarity with motifs from the TRANSFAC and JASPAR repositories [23,24], that is, the ChIP-seq data sets employed here are dominated by direct TF-DNA binding for the target TF (see http://factorbook.org). Indeed, all of the predictive known motifs listed in Table 1 were rediscovered through *de novo *motif analysis, with the exceptions of E2F4 and E2F6 (see also http://factorbook.org). In the cases of E2F4 and E2F6, even when the top-ranked *de novo*-discovered motif differed from those listed in Table 1, direct TF-DNA binding by the target TFs was indicated.

Note that, by default, POSSUM computes log-likelihood scores using local nucleotide abundances within a 100-bp window. Adjusting the size of this window had little effect upon the AUC computed for a given PWM (see Figure S8 in Additional file 1); the default 100-bp window size for local abundances was used throughout this work.

The predicted TF binding sites that resulted from scanning PWMs over ChIP-seq data sets were distributed across the human genome. Our functional tests, however, were carried out exclusively on promoter sequences from the library of SwitchGear Genomics. In selecting predicted TF binding sites for assaying biological function on promoters, we first restricted our predicted TFBS list to include only binding sites that overlapped with the SwitchGear library and applied a set of additional filters: the log-odds score from PWM scanning must be at least 10-fold greater than that of the background for our control set, and the false discovery rate reported for the ChIP-seq peak by MACS [97] must be less than 0.05. On average, the predicted TF binding sites were centered on the summits (point of maximum signal) from the ChIP-seq data (see Figure S9 in Additional file 1).

For each predicted TFBS that was functionally tested, mutations were chosen by mutating five nucleotides such that the binding site match to the PWM was minimized. By comparing the resulting mutated sequence to a library of known consensus binding sequences, we ensured that the TFBS was not mutated into a sequence that was favorable for binding another TF. Data from our TFBS predictions and measurements are available in Table 4 in Additional file 2 and will also be made available at the UCSC Genome Browser [100], for which an ENCODE page has been developed [101].

### Negative controls

Negative control experiments were performed to compare the activities of wild-type promoters with those of promoters mutated in regions with no expected TF binding. To locate regions on promoters with no expected TF binding, 'unbound' genomic locations with no measured ChIP-seq signal in any of the ENCODE Consortium data sets and no reported hypersensitivity to cleavage by DNase I (open chromatin) were tabulated (see Table S6 in Additional file 1 for a complete listing of data sets used to find experimentally unbound genomic regions). A negative control 'TFBS' (12 bp in width) was assigned at a location chosen randomly (using a uniform distribution) from within the resulting 'unbound' regions. These control 'TFBSs' were randomly mutated at five sites. At FDR < 0.025, we detected a single functional result from the 12 negative control binding sites that were assayed in K562 cells, representing a false positive rate of 8.3%.

### Functional tests of putative TF binding sites not bound in vivo

In addition to our negative controls, we functionally tested a different class of TF binding sites: sequences that were predicted to bind TFs based upon scanning with PWMs but were not observed to be bound *in vivo*. We tested 23 sequences that, like our negative controls, were located in 'unbound' genomic locations with no measured ChIP-seq signal in any of the ENCODE Consortium data sets and no reported hypersensitivity to cleavage by DNase I (open chromatin). Unlike our negative controls, however, these sequences were strong candidate TFBSs based upon matches to PWMs. These putative binding sites were identified based on motifs for CTCF(1), GATA2(2), MAX(1), NFY(1), STAT proteins (17), and USF2(1), where the numbers of binding sites tested for each motif are indicated in parentheses (see Table S6 in Additional file 1 for a complete listing of data sets used to develop the functional tests reported in this section). We assayed these TF binding sites on promoter constructs transiently transfected into K562 cells. At the FDR < 0.025 threshold, we detected function for GATA2(1), NFY(1), and STAT proteins (5), for an overall functional rate of 30%. This rate of functional detection is notably lower than that for the predicted TFBSs that were present within ChIP-seq peaks.

### Transient transfection assay

We systematically identified transcription start sites throughout the genome and have cloned more than 16,000 approximately 1 kb promoter fragments based on this start site information into a modified version of Promega's pGL4.11 firefly luciferase reporter vector. This clone collection became the starting material for site-directed mutagenesis using a modified version of the Quikchange protocol (Agilent Technologies, Inc., Santa Clara, CA, USA) [101]. All mutants were sequence confirmed and then re-arrayed alongside a wild-type control. Each mutant and accompanying wild-type was then mini-prepped three times to minimize the possibility that the variation between sample preparations would result in a significant difference between wild type and mutant (see Figure S10 in Additional file 1 for a schematic of our transient transfection assay).

We optimized transfection conditions for each cell line independently. The final conditions are described in Table S7 in Additional file 1. Irrespective of the cell line, the work flow was similar, save for the differences laid out in the supplementary table. In brief, after preparing a master mix containing 3.5 replicates worth of DNA and transfection reagent and incubating for the recommended amount of time, we added a quantity of freshly counted cells resuspended in warm, complete media sufficient for 3.5 replicates. After mixing thoroughly, we aliquoted the indicated volume into replicate white assay plates and placed at 37° for 24 h. Thus, each construct was transfected a total of nine times (three prep replicates each transfected three times). After incubation, the plates were removed, and SteadyGlo luciferase assay reagent (Promega Corporation, Madison, WI, USA) was added to each well. The plates were incubated in the dark for at least 30 minutes and then read on an LmaxII-384 luminometer (Molecular Devices, LLC, Sunnyvale, CA, USA).

### Statistical testing

The resulting luminosity data (three transfections, each with three prep replicates) were analyzed using *t *tests. A multiple testing correction was applied to the resulting *p *values via the Benjamini-Hochberg rule: in total, there were 1,855 hypothesis tests from 455 TFBSs tested in four cell lines, plus 12 negative control experiments in the K562 cell line and 23 experiments for putative TFBSs that were unbound *in vivo*, also in the K562 cell line. Experiments where FDR < 0.025 for the mutation were considered to demonstrate TFBS function. All other statistical comparisons (except where noted) for significant differences between distributions were carried out using the Kolmogorov-Smirnov test, which is appropriate for detecting differences in two distributions that may have similar means.

## Abbreviations

AUC: area under the receiver operating characteristic curve; ChIP-seq: chromatin immunoprecipitation with high-throughput sequencing; ENCODE: ENCyclopedia of DNA Elements; PWM: position weight matrix; TF: transcription factor; TFBS: transcription factor binding site; TSS: transcriptional start site.

## Competing interests

The authors declare that they have no competing interests.

## Authors' contributions

ZW conceived the work and supervised the analysis. TWW carried out the predictions and analysis. JW contributed to the predictions. PJC, ECP and SFA conducted the experiments. NDT and RMM supervised the experiments. The manuscript was written by TWW and ZW, with contributions from the other authors. All authors read and approved the final manuscript.

## Supplementary Material

Additional file 1 **Supplementary Tables S1 to S4 and Figures S1 to S10, in portable document format (pdf)**. Click here for file

Additional file 2 **Supplementary Table S5, data from luciferase assays, in tab-delimited text format**. Click here for file
