# Supplementary material for: Functional analysis of transcription factor binding sites in human promoters
Source: Genome Biol. 2012 Sep 5;13(9):R50. doi: 10.1186/gb-2012-13-9-r50 (PMC3491394; doi:10.1186/gb-2012-13-9-r50)
Supplement: Additional file 1 — Supplementary Tables S1 to S4 and Figures S1 to S10, in portable document format (pdf). [file gb-2012-13-9-r50-S1.pdf]

Functional analysis of transcription factor binding sites in human promoters: Supplementary material

Troy W. Whitfield,[1] Jie Wang,[1] Patrick J. Collins,[2] E. Christopher Partridge,[3]
Shelley Force Aldred,[2] Nathan D. Trinklein,[2] Richard M. Myers,[3] and Zhiping Weng[1]

[1]Program in Bioinformatics and Integrative Biology and Department of Biochemistry and Molecular Pharmacology, Worcester, MA 01605
[2]SwitchGear Genomics, Menlo Park, CA 94025
[3]HudsonAlpha Institute for Biotechnology, Huntsville, AL 35806

Supplementary tables

TABLE S1: For each TF that was part of our functional study, columns list binding motif logos and sources of PWMs used for making TFBS predictions.

TF	Motif	PWM
CTCF	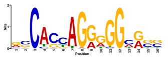	Ref. [31]
E2F4	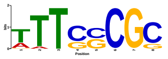	E2F.4 M00739
E2F6	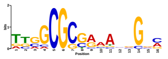	E2F.1 M00938
EGR1	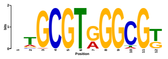	Egr.3 M00245
GABP	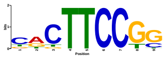	Ref. [103]
GATA1	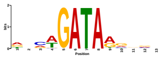	GATA.1 M00128
GATA2	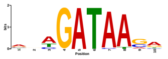	GATA.2 M00348
JUND	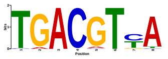	CREBP1 M00041
MAX	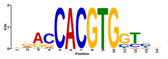	cMycMax M00118
STAT1	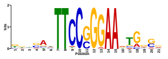	STAT1 M00224
USF1	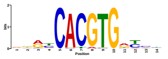	USF M00121
YY1	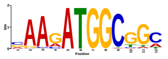	Ref. [103]

TABLE S2: Total RPKM (reads per kilobase of exon model per million mapped reads) levels for selected TF families in two cell lines. Where noted, RNA expression levels are summed for all members of each family with similar DNA binding specificities. For example, the STAT1 RPKM levels are for the sum of levels for STAT1, STAT3-4, STAT5A, STAT5B and STAT6. Data can be found in GEO, GSM765405 and GSM758575.

Transcription factor	K562[a]	HepG2[a]	K562[b]	HepG2[b]
CTCF	4.96	3.50	4.96	3.50
E2F4	15.44	17.34	30.88	30.03
E2F6	3.19	2.97	30.88	30.03
EGR1	2.69	1.27	2.73	1.29
GABP	4.21	1.27	4.21	1.27
GATA1	20.04	0.008	23.74	4.14
GATA2	3.53	0.89	23.74	4.14
JUND	14.59	11.97	14.59	11.97
MAX	3.55	2.51	3.55	2.51
STAT1	2.93	3.20	54.99	28.12
USF1	3.33	1.90	9.27	15.03
YY1	5.41	4.75	5.41	4.75

[a]Measured RPKM for each TF.
[b]RPKM summed across TF family.

TABLE S3: Co-localizing transcription factors from analysis of ChIP-seq data. Pairs of TFs listed above have overlapping signals in ChIP-seq experiments and, moreover, have a distance distribution between their binding sites that is significantly different from random ($p < 0.01$). Binding sites for all TF pairs are within 100 bp of one another.

TF	TF2
CTCF	AP-1, AP-2, E2F1, E2F4, EBF, GABP, GR, HNF4, LBP-1, MAX, MYC
	NF-Y, NRF-1, Novel3, Novel5, PU.1, Pax5, SP1, USF, ZNF143
E2F4	CTCF, EGR1, GABP, NF-Y, Novel5, PU.1, SP1, YY1, ZNF143
GABP	CTCF, E2F1, E2F4, EGR1, NF-Y, NRF-1, Novel5, SP1, ZNF143
STAT1	AP-1
YY1	E2F4, EGR1, NF-Y, Novel5, SP1, ZNF143

TABLE S4: Gene expression omnibus (GEO) accession numbers or directions to file downloads from the UCSC data coordination center (DCC) for ENCODE ChIP-seq data-sets used in TFBS predictions.

Transcription factor	Data-sets
CTCF	GSM822311, GSM822311
E2F4	P[a]/wgEncodeYaleChIPseqPeaksK562bE2f4.tagAlign.gz
E2F6	P[a]/wgEncodeYaleChIPseqPeaksK562bE2f6.tagAlign.gz
EGR1	GSM803414, GSM803434
GABP	GSM803524, GSM803356
GATA1	GSM467647
GATA2	GSM467648
JUND	GSM487425
MAX	P[a]/wgEncodeYaleChIPseqPeaksK562MaxV2.tagAlign.gz
STAT	P[a]/wgEncodeYaleChIPseqPeaksK562Ifna30Stat1.tagAlign.gz
	P[a]/wgEncodeYaleChIPseqPeaksK562Ifna6hStat1.tagAlign.gz
	P[a]/wgEncodeYaleChIPseqPeaksK562Ifna30Stat2.tagAlign.gz
	P[a]/wgEncodeYaleChIPseqPeaksK562Ifna6hStat2.tagAlign.gz
USF1	GSM803441
YY1	P[a]/wgEncodeYaleChIPseqPeaksK562bYy1.tagAlign.gz

[a]http://hgdownload.cse.ucsc.edu/goldenPath/hg18/encodeDCC/wgEncodeYaleChIPseq must be included in the path in order to download data.

TABLE S6: UCSC accession numbers for ENCODE ChIP-seq and DNase-seq data-sets (in K562 cells) used in predictions of random unbound control sequences and high-scoring unbound TFBS sequences.

Transcription factor	UCSC accession
ATF3	wgEncodeEH000700
BCLAF1	wgEncodeEH001571
BDP1	wgEncodeEH000678
BRF1	wgEncodeEH000679
BRF2	wgEncodeEH000767
BRG1	wgEncodeEH000724
CCNT2	wgEncodeEH001864
CFos	wgEncodeEH000619
Cjun	wgEncodeEH000673, wgEncodeEH000667, wgEncodeEH000668, wgEncodeEH000620
Cmyc	wgEncodeEH000659, wgEncodeEH000669, wgEncodeEH001867, wgEncodeEH000670, wgEncodeEH000621
CTCF	wgEncodeEH000042, wgEncodeEH000535, wgEncodeEH000399
E2F4	wgEncodeEH000671
E2F6	wgEncodeEH000676
FOSL1	wgEncodeEH001637
GABP	wgEncodeEH001604
GATA1	wgEncodeEH000638
GATA2	wgEncodeEH000683
GTF2b	wgEncodeEH000703
HEY1	wgEncodeEH001481
HMGN3	wgEncodeEH001863
INI1	wgEncodeEH000725
IRF1	wgEncodeEH001865, wgEncodeEH001866
JunD	wgEncodeEH001211
KAP1	wgEncodeEH001764
MAX	wgEncodeEH000637
NELFe	wgEncodeEH000701
NF-E2	wgEncodeEH000615
NF-YA	wgEncodeEH002021
NF-YB	wgEncodeEH002024
Nrf1	wgEncodeEH001796
PolII	wgEncodeEH000053, wgEncodeEH001581, wgEncodeEH001633, wgEncodeEH000555, wgEncodeEH000615
	wgEncodeEH000660, wgEncodeEH000661, wgEncodeEH000704, wgEncodeEH000662, wgEncodeEH000727
PolIII	wgEncodeEH000694
PU.1	wgEncodeEH001482, wgEncodeEH001482
Rad21	wgEncodeEH000649
RPC155	wgEncodeEH000680
SIN3A	wgEncodeEH001607
SIRT6	wgEncodeEH000681
SIX5	wgEncodeEH001483
SP1	wgEncodeEH001578
SRF	wgEncodeEH001600
SIX5	wgEncodeEH001483
STAT1	wgEncodeEH000663, wgEncodeEH000664, wgEncodeEH000760, wgEncodeEH000761
STAT2	wgEncodeEH000665, wgEncodeEH000666
TAF1	wgEncodeEH001582, wgEncodeEH001582
TAF7	wgEncodeEH001654
TBP	wgEncodeEH001825
TFIIIC-110	wgEncodeEH000748
THAP1	wgEncodeEH001655
USF1	wgEncodeEH001583
USF2	wgEncodeEH001797
XRCC4	wgEncodeEH000650
YY1	wgEncodeEH001584
ZBTB33	wgEncodeEH001569
ZBTB7A	wgEncodeEH001620
DNase I HS	wgEncodeEH000484

TABLE S7: Experimental conditions for transient transfection assays in four immortalized human cell lines

Cell line	ATCC No.	N_p [a]	N_c [b]	FuGene 6[c]	Lipofectamine LTX [c]	PLUS reagent[c]	DNA[d]	V[e]
K562	CCL-243	96	25000	0	0.4	0.1	100	100
HT1080	CCL-121	384	7500	0.3	0	0.1	50	75
HCT116	CCL-247	384	15000	0	0.35	0	50	75
HepG2	HB-8065	384	10000	0.6	0	0.1	100	75

[a]Number of plate wells
[b]Number of cells per well.
[c]In μL.
[d]In ng.
[e]Volume per well, in μL.

Supplementary figures

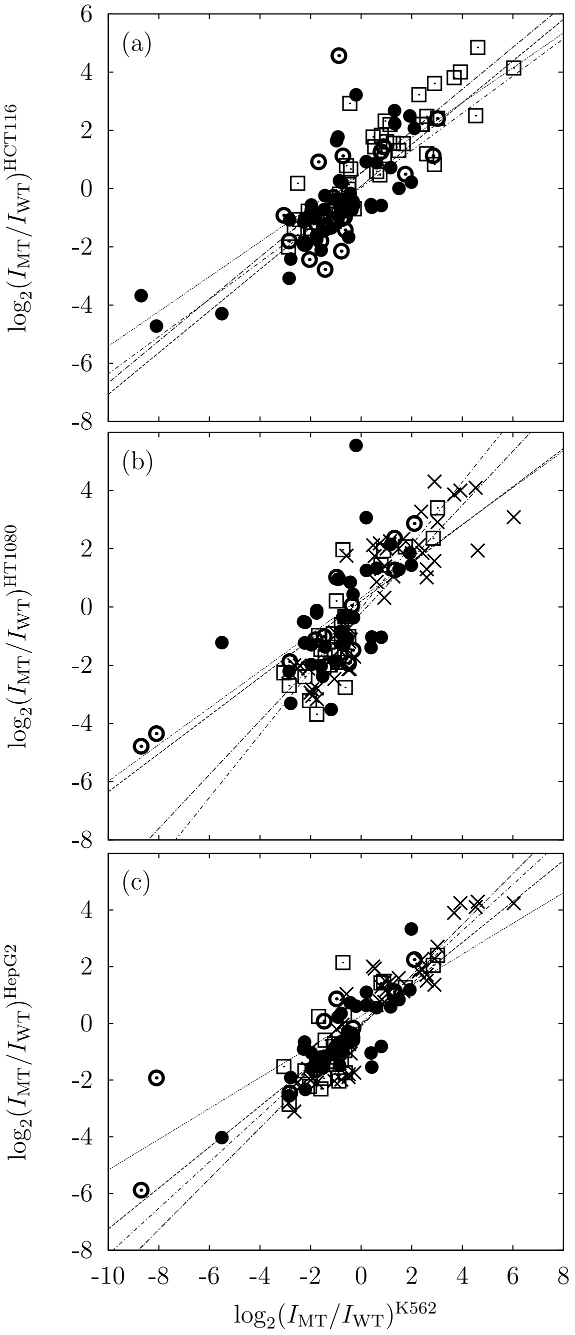

FIG. S1: Pairwise scatter plots for $\log_2(I_{\rm MT}/I_{\rm WT})$, where $I_{\rm MT}$ and $I_{\rm MT}$ are the mutant and wild-type normalized luminosities, respectively, in four cell lines (K562, HCT116, HT1080 and HepG2). Luminosities are plotted for CTCF ($\bullet$), GATA2 ($\odot$), STAT1 ($\boxdot$) and YY1 ($\times$), in each case for binding sites that were functionally verified in 3 or 4 cell lines. The slopes, m, for each linear regression above are as follows: (a) $m_{\rm CTCF} = 0.72{\pm}0.1$, $m_{\rm GATA2} = 0.60{\pm}0.1$, $m_{\rm STAT1} = 0.64{\pm}0.2$, $m_{\rm YY1} = 0.72{\pm}0.05$ (b) $m_{\rm CTCF} = 0.65{\pm}0.2$, $m_{\rm GATA2} = 0.63{\pm}0.1$, $m_{\rm STAT1} = 1.08{\pm}0.1$, $m_{\rm YY1} = 0.92{\pm}0.07$ (c) $m_{\rm CTCF} = 0.72{\pm}0.1$, $m_{\rm GATA2} = 0.54{\pm}0.1$, $m_{\rm STAT1} = 0.81 \pm 0.1$, $m_{\rm YY1} = 0.89 \pm 0.05$.

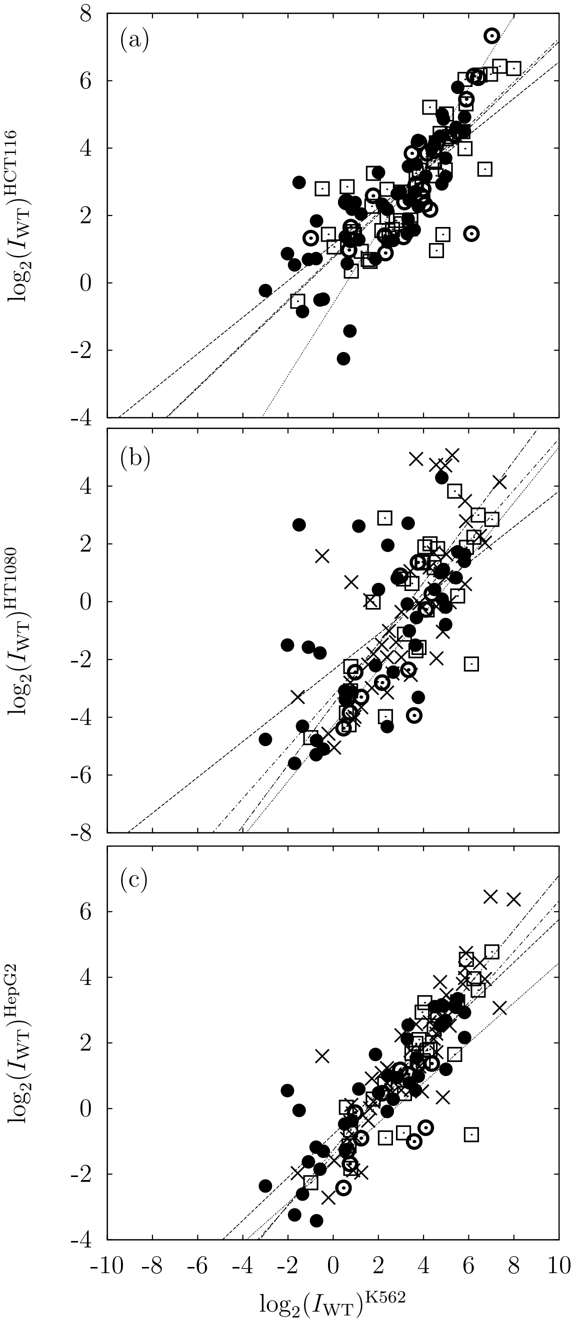

FIG. S2: Pairwise scatter plots for $\log_2(I_{\rm WT})$, where $I_{\rm MT}$ is the wild-type normalized luminosity, in four cell lines (K562, HCT116, HT1080 and HepG2). Luminosities are plotted for CTCF ($\bullet$), GATA2 ($\odot$), STAT1 ($\boxdot$) and YY1 ($\times$), in each case for binding sites that were functionally verified in 3 or 4 cell lines. The slopes, m, for each linear regression above are as follows: (a) $m_{\rm CTCF} = 0.54 \pm 0.06$, $m_{\rm GATA2} = 1.06 \pm 0.27$, $m_{\rm STAT1} = 0.64 \pm 0.1$, $m_{\rm YY1} = 0.65 \pm 0.3$ (b) $m_{\rm CTCF} = 0.62 \pm 0.2$, $m_{\rm GATA2} = 0.96 \pm 0.3$, $m_{\rm STAT1} = 0.89 \pm 0.2$, $m_{\rm YY1} = 1.05 \pm 0.1$ (c) $m_{\rm CTCF} = 0.65 \pm 0.06$, $m_{\rm GATA2} = 0.61 \pm 0.1$, $m_{\rm STAT1} = 0.77 \pm 0.1$, $m_{\rm YY1} = 0.84 \pm 0.06$.

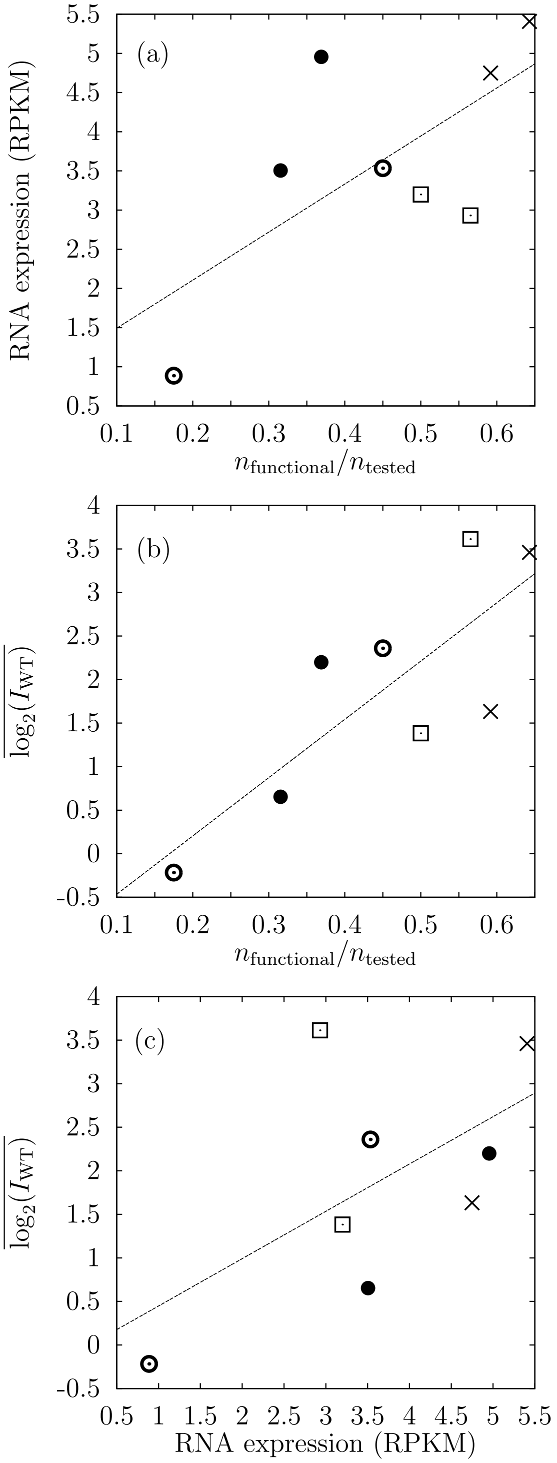

FIG. S3: Pairwise scatter plots for $\log_2(I_{\mathrm{WT}})$, $n_{\mathrm{functional}}/n_{\mathrm{tested}}$, and RNA expression (data can be found in GEO: GSM765405, GSM758575), where n is the number of TF binding sites and I_{MT} is the wild-type normalized luminosity in the K562 and HepG2 cell lines. Points are plotted for CTCF ($\bullet$), GATA2 ($\odot$), STAT1 ($\boxdot$) and YY1 ($\times$), in each case for binding sites that were functionally verified in 3 or 4 cell lines. The Pearson's correlation coefficients are (a) $r = 0.67$, (b) $r = 0.8$ and (c) $r = 0.59$.

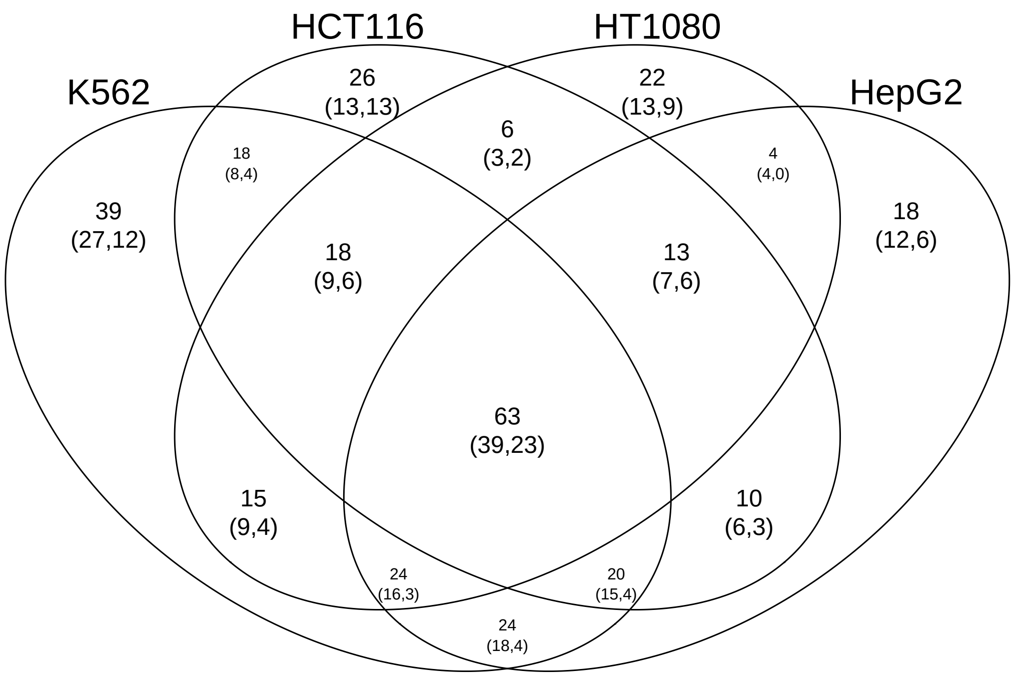

FIG. S4: Venn diagram for functionally verified (FDR < 0.025) TFBS binding sites in four different cell lines. There were 135 TF binding sites that were tested but not functional in any of the four cell lines.

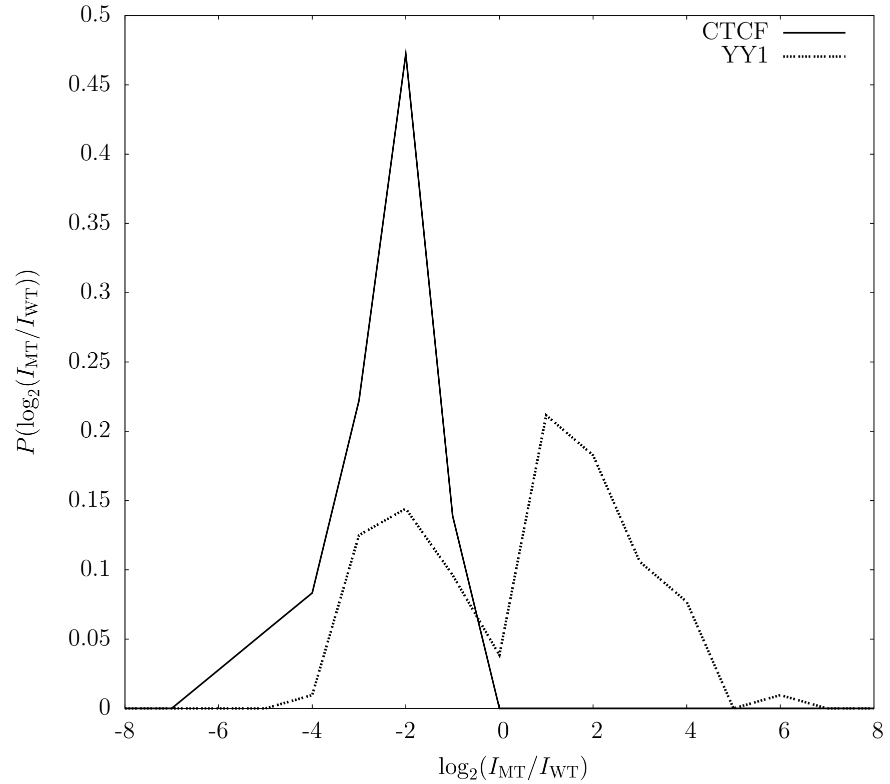

FIG. S5: Probability distributions for signal, $\log_2(I_{\rm MT}/I_{\rm WT})$, of ubiquitously functional CTCF (solid line) and YY1 (dashed line) binding sites.

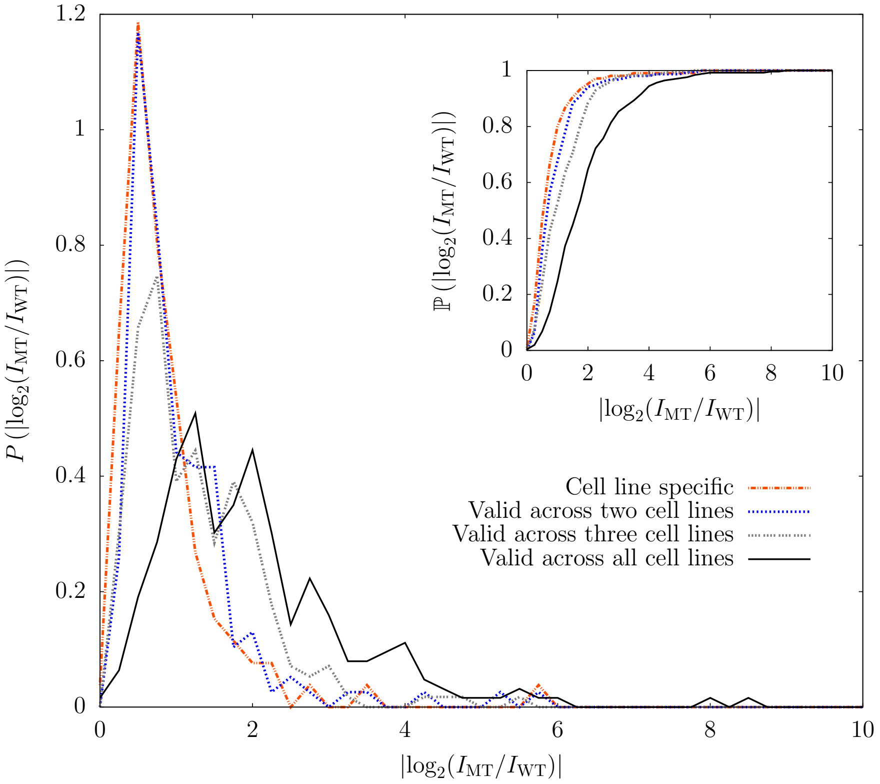

FIG. S6: Distinguishing between TF binding sites that were functionally validated in different cell lines using $|\log_2(I_{\rm MT}/I_{\rm WT})|$. Plotted in the inset is the cumulative probability, $\mathbb{P}(x) = \int_0^x P(x')dx'$, where $x = |\log_2(I_{\rm MT}/I_{\rm WT})|$. The means of $P(|\log_2(I_{\rm MT}/I_{\rm WT})|)$ are 0.97, 1.16, 1.41 and 2.16 (validation in one, two, three and four cell lines, respectively).

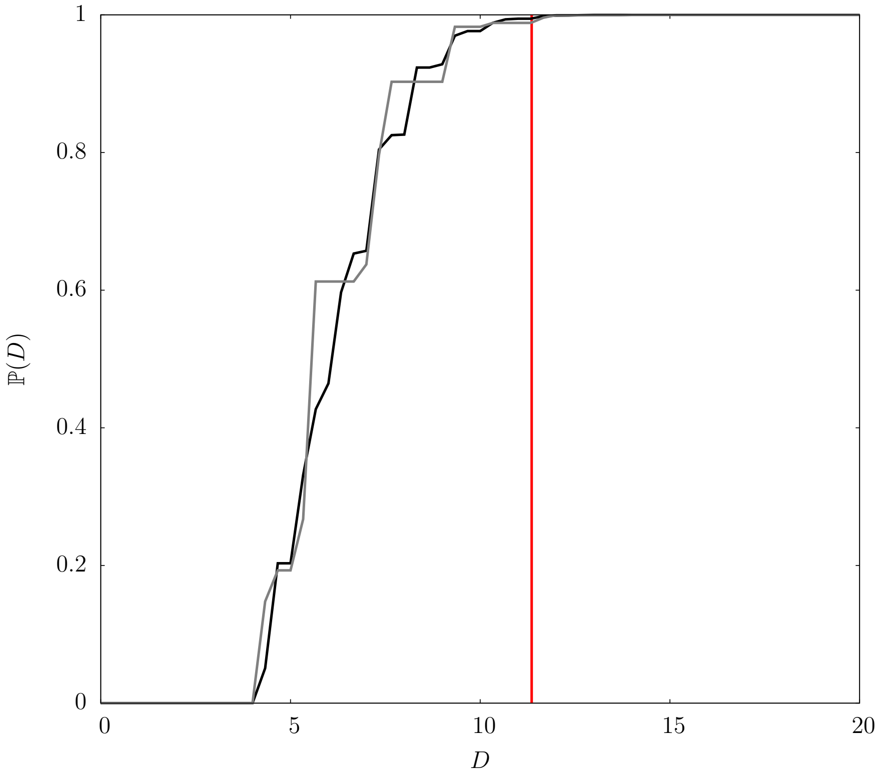

FIG. S7: Statistical significance of the observed difference in activating and repressive classes of YY1 motifs at position 4. The cumulative distribution function, $\mathbb{P}(D) = \int_0^D P(D')dD'$, is plotted where the test statistic, $D = \sqrt{(n_1^A - n_2^A)^2 + (n_1^C - n_2^C)^2 + (n_1^G - n_2^G)^2 + (n_1^T - n_2^T)^2}$ is the Euclidean distance between the ends of radius vectors for two PWMs, 1 and 2, at a specific position (position 4). Our observed motifs, Fig. 2 (b) and (c) were derived from groups of 9 (ubiquitously activating) and 16 (ubiquitously repressing) TFBS instances, respectively. Accordingly, we have estimated $\mathbb{P}(D)$ by comparing 2×10^6 groups of 9 and 16 TFBSs, randomly drawn from YY1 binding sites that were ubiquitously validated (grey line) or tested (black line). The red vertical line indicates the calculated D from our observations (Fig. 2). We estimate $p < 0.005$ when all tested YY1 binding sites are considered and $p < 0.012$ when only ubiquitously validated sites are considered.

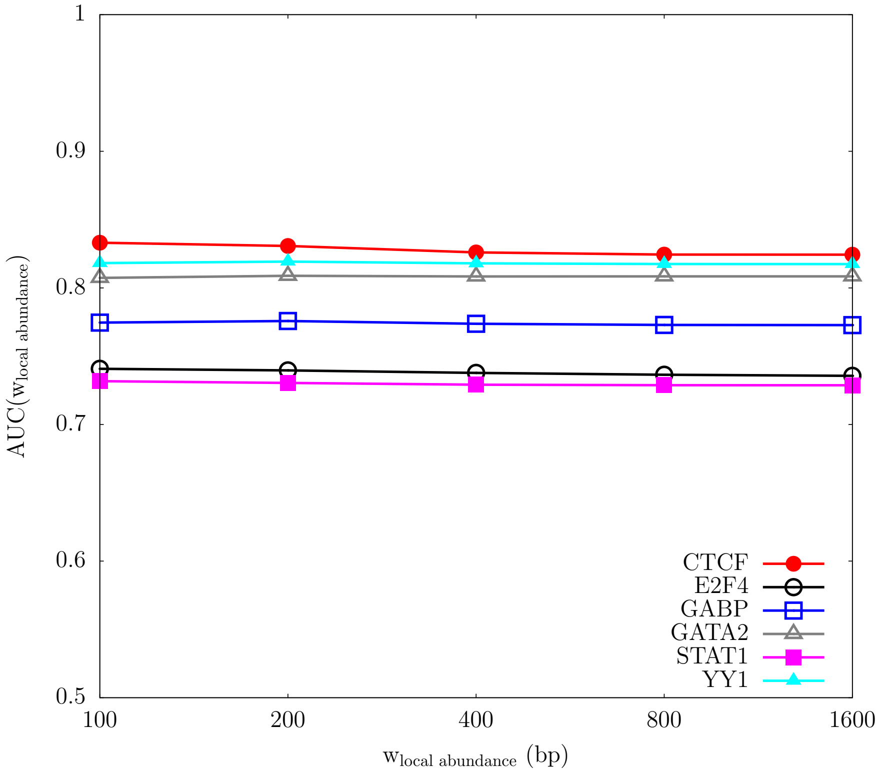

FIG. S8: Area under the receiver operating characteristic curve (AUC) as a function of window size for determining local nucleotide abundances.

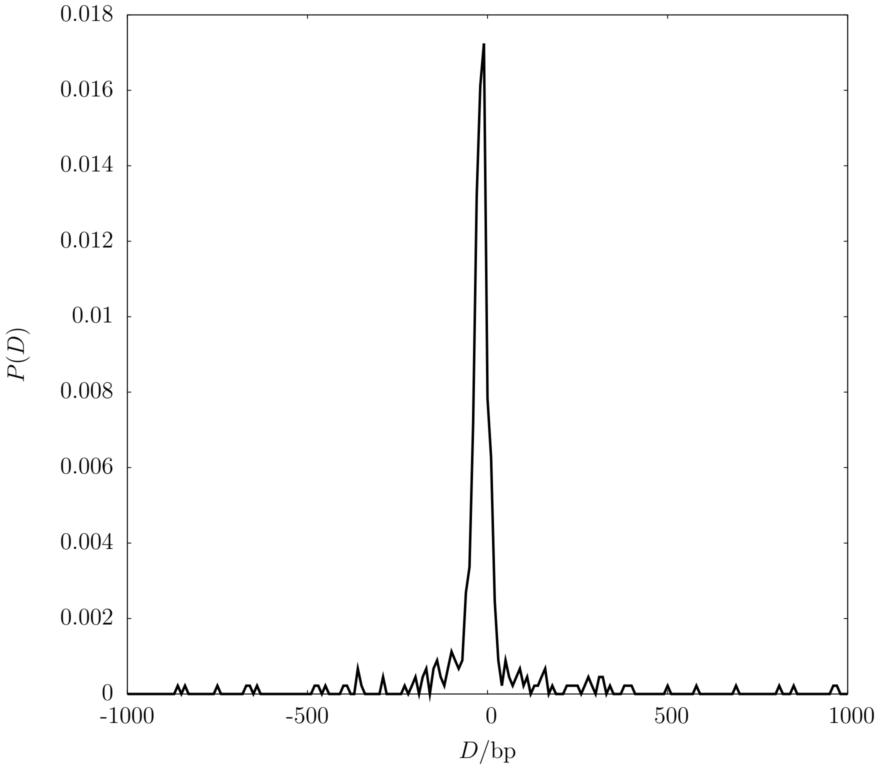

FIG. S9: Probability distribution of distances, D, in base pairs between predicted TFBSs and the nearest position of maximum ChIP-seq read density (i.e. the summit of each "peak").

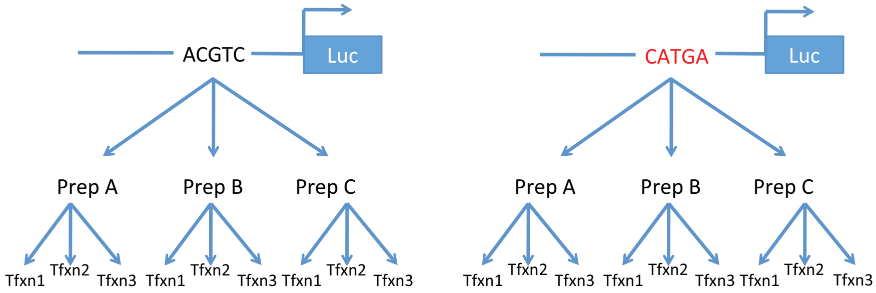

FIG. S10: Schematic of transient transfection assays that were carried out in wild type and mutant constructs.
